# Beyond the Material: Engineering Sustainable Soft Robots and Electronics

**DOI:** 10.1002/advs.74485

**Published:** 2026-02-16

**Authors:** Florian Hartmann, Martin Kaltenbrunner

**Affiliations:** ^1^ Biomimetic Materials and Machines Group Max Planck Institute for Intelligent Systems Stuttgart Germany; ^2^ Division of Soft Matter Physics Institute for Experimental Physics Johannes Kepler University Linz Austria; ^3^ Soft Materials Lab Linz Institute of Technology Johannes Kepler University Linz Austria

Research on sustainable materials in soft robotics and electronics is essential to developing eco‐friendly technologies that reduce environmental impact. As these fields grow, the demand for flexible, adaptable materials that do not contribute to long‐term waste or pollution is increasing. Across biodegradable, recyclable, and edible material platforms, recent advances are beginning to align device performance with circular‐economy principles, minimizing reliance on non‐renewable resources while addressing the end‐of‐life challenge of electronic and robotic waste. This special issue captures these efforts, spanning materials, fabrication, devices, and deployment concepts that collectively point toward high‐tech systems that are innovative during use and responsible after use.

This special issue comprises 20 Research Articles, 8 Reviews, and 1 Perspective. The contributions cover biodegradable and edible structural materials; biohybrid and nature‐sourced building blocks; scalable and data‐driven fabrication methods; bioinspired actuators; biodegradable sensing platforms and adaptive robotic skins; transient semiconductors and recyclable electronics; bio‐based energy harvesting and edible energy storage; and broader frameworks that connect material degradation and system design to the realities of deployment in natural environments.

Qilin Hua, Guozhen Shen, and co‐authors provided a general overview of the progress and challenges in bringing soft robots closer to real‐world scenarios (article number 202506296). They reviewed bioinspired intelligent soft robotics from actuation and programmable materials to manufacturing and control, highlighting the key challenges that remain when intelligent behavior must be achieved.

Several papers at the foundation of soft robotics and electronics advanced sustainable material platforms and substrates for soft systems. Guoyong Mao, Enbo Xu, and co‐authors presented a phase‐separation strategy of starch induced by antisolvents to enable elastic, edible hydrogels with tunable strength and extensibility. They used the material to demonstrate a pneumatic soft gripper that biodegraded in soil within a few days, presenting a route toward biodegradable yet durable soft robots (article number 202507216). For the use in digital light processing printing, Dorothea Helmer, Edoardo Milana, and co‐authors developed a bio‐based resin made from acrylated epoxidized soybean oil. Using this material and fabrication method, they demonstrated monolithic, origami‐based actuators and grippers driven by vacuum (article number 202520529). Addressing life cycle aspects and material reuse, Yu Jun Tan and co‐authors repurposed waste from kombucha bacterial cellulose through mild purification to form compostable films with improved strength. Furthermore, they introduced a method for patterning conductive tracks, enabling a biodegradable pressure‐sensing electronic device (article number 202514521). Kunal Masania and co‐authors focused on manufacturing biomaterials and developed a strategy to enhance ultrasonic wood welding by introducing 3D‐printed lignin energy directors. This approach increased interfacial lignin fusion and improved durability in wet conditions, offering a scalable pathway to stronger wood joints with potential multifunctionality (article number 202507055).

Biohybrid approaches broaden the sustainable design space beyond the use of biopolymers to make materials from cell‐based materials and structures. Josie Hughes and co‐authors demonstrated how discarded langoustine abdomen exoskeletons can be repurposed as jointed, load‐bearing actuators that enable grasping, high‐speed bending, and swimming. They also proposed a cyclic design strategy grounded in bio‐waste reuse (article number 202517712). Ritu Raman and co‐authors introduced modular biohybrid muscle‐tendon units that couple engineered skeletal muscle with tough, adhesive hydrogel tendons. By tuning stiffness and pretension, they improved force transmission and reported an approximately 11‐fold increase in power‐to‐weight ratio with durability beyond 7000 cycles (article number 202512680). Looking to plants as renewable robotic matter, Jun Shintake and co‐authors discussed how plant movement and inherent multifunctionality could form the basis for biodegradable robots and defined research directions for this emerging field (article number 202512896).

A second set of contributions addressed fabrication processes and design methodologies that help translate sustainable materials into more robust devices. ChangKyu Yoon, Jun Dong Park, and co‐authors created a rheology–printability database of 150 hydrogels and developed a machine‐learning model that predicted the print fidelity of direct ink writing with an accuracy of 10%. This model clarified which nonlinear rheological features govern horizontal versus vertical printability (article number 202507639). Hortense Le Ferrand and co‐authors reviewed how bioinspired microstructures can enhance sensing and actuation performance, as well as support the integration of sensing, actuation, and computation in robotic materials (article number 202509739).

In soft systems, adhesion and bonding mechanisms replace screws for assembly and interfacing with natural soft bodies or environments. Suk‐Won Hwang and co‐authors reviewed biodegradable adhesive systems for bio‐integrated applications. They organized advances by adhesion mechanism and outlined routes to strong wet adhesion, degradation, and multifunctional integration with bioelectronics (article number 202512633). Yu Tian, Hongbo Zeng, and co‐authors showed that a polyacrylamide hydrogel layer can transition from low friction behavior to ultra‐high friction and adhesion during dehydration due to volumetric shrinkage and increased interfacial interactions. This approach enabled high, MPa‐level adhesion strengths across surfaces, reframing wet‐to‐dry dynamics as a functional mechanism for soft robotic adhesion (article number 202507827).

In bioinspired actuation, multiple works demonstrated how performance, programmability, and durability can be improved through materials and process development. Mahdi Bodaghi, Mostafa Baghani, and co‐authors reviewed 4D printing of magnetically responsive shape memory polymer composites. They compared key additive manufacturing routes and examined the effects of processing methods, such as melt mixing versus solvent casting, on filler dispersion, mechanics, and actuation performance (article number 202513091). Yingxiang Liu and co‐authors developed twisted artificial muscles inspired by plant twinning. They added a bioinspired insulation layer to reduce underwater heat loss and integrated rapid elastic energy release to drive a bionic ray with improved aquatic locomotion (article number 202507572). Cong Zhang, Qingyu Peng, Xiaodong He, and co‐workers achieved additive‐free Ti_3_C_2_T_x_ MXene‐based actuators through the gradient assembly of nanosheets of various sizes and a cyclic low‐temperature annealing–rehydration method. This process yielded programmable, moisture‐driven deformation with high‐humidity stability over extended actuation cycles (article number 202510243). Longfei Chang, Qingyu Peng, Ying Hu, and co‐authors reported on a moisture‐driven MXene/carboxymethyl chitosan actuator fabricated via tilt filtration that exhibited diode‐like unidirectional bending. These actuators enabled metabolism‐driven devices, including oscillators, crawling robots, and respiratory sensors (article number 202507845).

For soft and flexible sensors, strategies for the end of use and design methodologies can be developed alongside the use of sustainable materials to improve sensor performance or efficacy in diverse applications, from tactile sensing to in‐vivo use. Aihua Zhong, Long‐Biao Huang, and co‐authors reviewed biodegradable materials for sensors for soft robots, discussing challenges in degradation control and scalable integration. They systematically covered material classes and sensing modalities, including strain/pressure and tactile sensing for applications in implantable electronics and environmental monitoring (article number 202510320). Yingying Zhang, Wei Fan, and co‐authors reviewed top‐down fabricated, wood‐derived pressure and strain sensors. They analyzed how wood composition and hierarchical microstructure influenced sensing mechanisms, while emphasizing process efficiency and reduced energy consumption (article number 202507712). Filipe Arroyo Cardoso, Clementine M. Boutry, and co‐authors reviewed wearable and implantable muscle‐monitoring devices, focusing on innovations in materials and devices across electrophysiology, biomechanics, and oxygenation sensing for continuous monitoring beyond the clinic (article number 202509934). David Hardman and co‐authors developed an automated benchmarking platform that compared 15 identically shaped soft robotic fingertips spanning multiple materials and sensing mechanisms. This platform enabled task‐optimized selection and self‐configuration of grippers across environments and lifetimes (article number 202509991). Hedan Bai and co‐authors developed a method to allow dynamic bond exchanges in silicones. This method allowed them to demonstrate a robotic skin that combined shape adaptation, self‐healing, mechanically programmable sensing, and on‐demand destructibility, offering a single‐material route to adaptable tactile interfaces with controllable lifetime (article number 202508823).

Transient and edible electronics extend sustainability to active components and semiconductors, where end‐of‐life management often remains challenging. Alessandro Luzio, Mario Caironi, and co‐authors reported on a solution‐based deposition method for porous “Pigment Blue” films that enabled low‐voltage, edible, electrochemical transistors. This method included devices made from pigment extracted from toothpaste, which highlights recycling‐compatible sourcing (article number 202416141). Jong‐hyoung Kim, Jae‐Young Bae, Seung‐Kyun Kang, and co‐authors established a biodegradable semiconductor platform using Mg_2_Si thin films. They systematically investigated the hydrolysis behavior of Mg_2_Si across ionic and pH conditions, confirmed cytocompatibility, and demonstrated integration into thermoelectric harvesters and near‐infrared photosensors (article number 202518093). Clementine M. Boutry and co‐authors engineered biodegradable magnetocaloric wax composites based on Mn_0.65_Fe_1.30_P_0.65_Si_0.37_ with a tuned Curie temperature near 43°C. This composite enabled self‐limiting, burn‐safe magnetothermal heating and melting under alternating magnetic fields for biomedical use (article number 202509914). Their work also demonstrated cytocompatibility and controlled degradation, which supported the development of temperature‐regulated therapeutic and delivery systems.

Research on sustainable soft robotics and electronics often overlooks energy storage and harvesting. Here, these topics are addressed with food‐safe chemistries and bio‐based electrification interfaces. Bokeon Kwak, Dario Floreano, and co‐authors presented an edible pneumatic battery and valve system that is driven by citric acid–bicarbonate neutralization. This system enabled regulated CO_2_ generation for sustained and repeated pneumatic actuation in fully edible robotic demonstrations (article number 202509350). Behnam Kamare, Fabian Meder, and co‐authors demonstrated that natural waxes can achieve energy‐harvesting of droplet impact with outputs comparable to fluoropolymer benchmarks. They implemented a modular, biodegradable harvester architecture that multiplied energy conversion events by guiding droplet motion (article number 202515266).

Finally, the articles in this issue also emphasized the importance of biodegradability when deploying robots and sensors in natural ecosystems, for example, for environmental monitoring. Andrew K. Schulz, Florian Hartmann, and co‐authors introduced an ecosystem‐centered robot design framework that frames biodegradation as context‐dependent and provided principles that connect ecosystem conditions, access constraints, and end‐of‐life strategies (article number 202509194). Ming Dong, Dimitrios G. Papageorgiou, and co‐authors developed edible and recyclable films from a gelatin/activated‐charcoal bilayer that supported multimodal strain, humidity, and temperature sensing. They demonstrated the use of these sensors for respiration monitoring and environmental monitoring, as well as soil degradation and water‐based recycling (article number 202507950). For distributed environmental sensing, Stefano Mariani, Barbara Mazzolai, and co‐authors introduced a degradable, seed‐inspired flyer that integrated cellulose nanocrystal aerogel sensors with edible dyes for colorimetric pH and ammonia detection. This design enabled passive dispersal and remote, image‐based monitoring with staged degradation (article number 202508949).

In summary, the contributions in this special issue demonstrate that mitigating the environmental impact of soft robotics and electronics necessitates more than simply replacing individual materials. Rather, it demands the design of devices, manufacturing processes, and deployment strategies that balance performance with recyclability, biodegradability, and responsible end‐of‐life approaches. These advances will accelerate the development of sustainable technologies in other application areas and fields that benefit from transient, environmentally friendly, and circular technologies. Finally, we thank all the authors who contributed to this special issue through their rigorous work and commitment to advancing sustainable soft technologies. We are most grateful to the editorial team of Advanced Science, particularly Dr. Hashini Thirimanne and Dr. Kirsten Severing, for their invaluable support in shaping this special issue and for their professional editing.

